# Bacterial and viral etiology of acute respiratory infection among the Forcibly Displaced Myanmar Nationals (FDMNs) in fragile settings in Cox’s Bazar- a prospective case-control study

**DOI:** 10.1371/journal.pntd.0011189

**Published:** 2023-04-10

**Authors:** Abu Bakar Siddik, Nabid Anjum Tanvir, Golam Sarower Bhuyan, Md. Shahariar Alam, Zahirul Islam, Md. Rakibul Hassan Bulbul, Md. Moniruzzaman, Charls Erik Halder, Tayabur Rahman, Hubert Endtz, Shakeel Ahmed, Firdausi Qadri, Valentina Sanchez Picot

**Affiliations:** 1 Institute for developing Science and Health initiatives, Dhaka, Bangladesh; 2 International Centre for Diarrhoeal Disease Research, Bangladesh, Mohakhali, Dhaka, Bangladesh; 3 International Organization for Migration(IOM), Cox’s Bazar, Bangladesh; 4 Refugee Health Unit, Office of the Refugee Relief and Repatriation Commissioner, Cox’s Bazar, Bangladesh; 5 Department of Medical Microbiology and Infectious Diseases, Erasmus MC, Rotterdam, Netherlands; 6 Bangladesh Institute of Tropical and Infectious Diseases, Chittagong, Bangladesh; 7 Acute Respiratory Infections Research & Interventions, Mérieux Foundation, Lyon, France; Pontificia Universidad Catolica de Chile, CHILE

## Abstract

The leading infectious cause of death in children worldwide is lower acute respiratory infection (LARI), particularly pneumonia. We enrolled a total of 538 acute respiratory infection (ARI) cases according to WHO criteria and age-sex matched 514 controls in the Forcibly Displaced Myanmar National (FDMN) refugee camps in Cox’s Bazar, Bangladesh, between June 2018 and March 2020 to investigate the role of bacteria, viruses, and their co-infection patterns and observe *Streptococcus pneumoniae* (*S. pneumoniae*) serotype distribution. According to the etiological findings, children ≤5 years of age have a higher bacterial positivity (90%) and viral positivity (34%) in nasopharyngeal samples (NPS) compared to those >5 years of age, in both ARI cases as well as for the control group. Among the bacteria, *S. pneumoniae* was predominant in both cases and controls (85% and 88%). Adenovirus (ADV)(34), influenza virus A and B (IFV-A, B)(32,23), and respiratory syncytial virus (RSV)(26) were detected as the highest number among the viruses tested for the ARI cases. The total number of viruses was also found higher in ≤5 years of age group. Within this group, positive correlation was observed between bacteria and viruses but negative correlation was observed between bacteria. Both single and co-infection for viruses were found higher in the case group than the control group. However, co-infection was significantly high for *Streptococcus aureus* (*S. aureus*) and *Haemophilus influenzae b* (*H. influenza b*) (p<0.05). Additionally, semi-quantitative bacterial and viral load was found higher for the ARI cases over control considering Cycle threshold (Ct)≤30. Pathogen identification from blood specimens was higher by qRT-PCR than blood culture (16% vs 5%, p<0.05). In the *S. pneumoniae* serotype distribution, the predominant serotypes in ARI cases were 23F, 19A, 16F, 35B, 15A, 20 and 10F, while 11A, 10A, 34, 35A and 13 serotypes were predominant in the control group. Pathogen correlation analysis showed RSV positively correlated with human metapneumovirus (HMPV), *S. aureus* and *H. influenza b* while *S. pneumoniae* was negatively correlated with other pathogens in ≤5 years age group of ARI cases. However, in >5 years age group, *S. aureus* and *H. influenza b* were positively correlated with IFVs, and *S. pneumoniae* was positively correlated with HMPV and ADV. Logistic regression data for viruses suggested among the respondents in cases were about 4 times more likely to be RSV positive than the control. Serotype distribution showed 30% for PCV10 serotypes, 41% for PCV13 and 59% for other serotypes. Also, among the 40 serotypes of *S. pneumoniae* tested, the serotypes 22F, Sg24, 9V, 38, 8, and 1 showed strong positive correlation with viruses in the case group whereas in the control group, it was predominant for serotypes 14, 38, 17F and 39 ARI cases were prevalent mostly in monsoon, post-monsoon, and winter periods, and peaked in September and October. Overall these region-specific etiological data and findings, particularly for crisis settings representing the FDMNs in Cox’s Bazar, Bangladesh, is crucial for disease management and disease prevention control as well as immunization strategies more generally in humanitarian crisis settings.

## Author summary

Acute respiratory infections (ARIs) are one of the most common communicable diseases in crisis settings, causing significant morbidity and mortality. Non-comparable as well as little aetiological data from refugee camps and surveillance, make it difficult to compare the prevalence of ARI disease between crisis and non-crisis contexts. In the world’s largest refugee settlement situated in Cox’s Bazar, Bangladesh, very little is known about the etiology of ARIs. To our best knowledge, this is the first case-control prospective study to evaluate the level of involvement of each etiological agent in the onset of LARI etiology, and the association between respiratory viral infections and invasive pneumococcal infections, *S. pneumoniae* serotype distribution, antimicrobial resistance pattern both in case and control group. Our study showed that ≤5 years age group was the most vulnerable, having the highest number of bacteria as well as viruses both in the case and control group. *S. pneumoniae* was the predominant bacteria, whereas, among the viruses ADV, IFVs, and RSV were predominant. Semi-quantitative bacterial as well as viral load was found higher in the case group. Positive correlation between viruses and other pathogens was higher in cases of RSV than in the control group. However, single as well as co-infection between viruses were significantly high among the ARI case while for bacteria it was comparable between the case and control group. ARI case enrollment peaked in September and October in the FDMN camps in Bangladesh.

## Introduction

Pneumonia, a severe form of lower acute respiratory infection, continues to be a major cause of death among children under the age of five years globally and accounted for 14% of deaths in this age group in 2019 [1]. Both children and adults are affected by pneumonia, but mortality is highest in South Asia and Sub-Saharan Africa [1]. Pneumonia infections can be caused by bacteria, viruses, fungi, and parasites. However, *S. pneumoniae*, *H. influenzae b* and *Staphylococcus aureus* are the most commonly found bacteria associated with acute respiratory infections (ARI), particularly pneumonia [2, 3]. Viruses associated with ARIs are Respiratory syncytial virus (RSV), influenza A virus (IFV-A) and influenza B virus (IFV-B), Human Parainfluenza Viruses 1, 2, 3 (HPIV1-3), adenovirus (ADV), and Human metapneumovirus (HMPV) are known to be a primary cause of ARI in children, as they can be attributed to over 60% of ARIs [4–6]. Several factors such as overcrowding, cold weather, and insufficient shelter all create an ideal condition for respiratory droplet transmission. Malnutrition, lack of sufficient sanitation, and stress can play a role in illness progression [1, 7]. The clinical symptoms of viral and bacterial pneumonia are very similar irrespective of causative agents [8] which creates a challenge in diagnosing and managing patients with ARIs making it difficult for physicians to determine the best course of treatment, which is based solely on clinical presentation, can lead to the overuse or misuse of drugs. Another major issue around the world is the rise of drug-resistant bacteria as well as the lack of evidence of clinical benefit. Data on the causative agents, improved and accessible diagnostic techniques and the drug sensitivity/resistance spectrum of the organisms are critical for optimal prevention and treatment of ARIs and lack of these data leads to irrational and inappropriate antimicrobial use, resulting in an increase in multi-drug resistant bacteria [9, 10]. Studies have documented the detection of respiratory infections that cause pneumonia throughout the previous decade, with wide variations in prevalence and pathogen spectrum seen among countries and regions, community demography, years, and seasons [11–14]. However, in everyday primary health care especially in refugee settings little is understood about the etiology of ARIs due to lack of data. Although it is difficult to compare the disease burden of ARIs in crisis and non-crisis settings due to data incompatibility, published evidence primarily from refugee camps and surveillance, suggests a very high morbidity and mortality (20-35%) percentage proportional mortality) as well as case fatality (up to 30-35%) due to ARIs [15]. Furthermore, data on pathogen detection in both symptomatic patients and healthy controls (without pneumonia) to differentiate between asymptomatic carriage and the presence of agents causing symptoms is also limited. Of bacterial pathogens responsible for ARIs. *S. pneumoniae* is a major pathogen causing pneumonia related deaths globally [2, 16, 17]. At least 98-100 pneumococcal capsular serotypes have been identified to date [18, 19]. Currently, pneumococcal conjugate vaccines (PCV) are being used to prevent *S. pneumoniae* infections (PCV10 and PCV13). These vaccinations only protect against a limited number of pneumococcal serotypes and do not protect against non-vaccine serotypes or unencapsulated *S. pneumoniae*. It is believed that the number of antibiotic-resistant non-vaccine serotypes has risen dramatically. Innovative, effective, and economical pneumococcal vaccines that can cover a wide range of serotypes are urgently needed. Therefore, to minimize respiratory infection-related morbidity and mortality, continuous monitoring and updates on etiology, better diagnostics, and serotype distribution, antibiotic resistance profiling is required. However, to the best of our knowledge, there have not been investigations on bacterial and viral etiologies of ARIs and *S. pneumonia* serotype distribution patterns in crisis settings, particularly in the Forcibly Displaced Myanmar Nationals (FDMNs). Nowadays, traditional culture methodologies are applied in limited resource settings. Although culture remains the gold standard, it has significant drawbacks, including specimen collection and transport requirements, the danger of pathogen growth inhibition due to recent antibiotic treatment, and long time needed to obtain results [20, 21]. Multiplexed molecular assays have recently emerged as a comprehensive diagnostic solution, providing laboratories with the necessary turnaround duration, sensitivity and specificity, and breadth of coverage of respiratory infections to support the physician’s decision-making needs [21]. To properly manage ARI cases, country-specific etiological and microbial sensitivity/resistance spectrum data are required. In Bangladesh, no study has been carried out in the FDMN camp settings focusing on pneumonia etiology. This study was driven by the understanding that ARI particularly pneumonia causes high mortality, particularly in crisis situations, and it intends to give data on ARI etiology. So, community-representative population studies are needed to better understand the true incidence of ARI infection. Moreover, the current new coronavirus disease (COVID-19) outbreak, which was first reported at the end of 2019 [22], is a public health emergency of international concern, prompting a growing interest in respiratory tract infections.

In the humanitarian crisis site of the FDMN in Cox’s Bazar, Bangladesh, no data is available regarding the presence of respiratory pathogens in ARI cases or in asymptomatic or healthy control persons as well as their age-specific distribution, co-infection frequency, and *S. pneumoniae* serotype distribution, etc. It is already known that numerous pathogens naturally colonize the respiratory tract [23], which explains why determining the true etiology of ARI is challenging. Moreover, organisms detected in the control group and their causal role cannot be excluded, as the human respiratory tract is a reservoir for many organisms, and their association with other organisms and their co-infection pattern could have resulted in the development of disease conditions [23]. The present case-control study aimed to gather evidence on the most common causative bacterial and viral agents of ARIs, the highest-risk age group in the FDMN camp settings. Considering the natural colonizing ability of the respiratory pathogens, healthy individuals (without ARI signs and symptoms) were enrolled to estimate the proportion of ARI cases attributable to detected viral and bacterial pathogens in the patients in a more accurate manner.

## Materials and methods

### Ethics statement

The study was approved by the ethical review committee, that is the National Research Ethics Committee (NREC) of the Bangladesh Medical Research Council (BMRC) [Registration number: 084 04 12 2017] and the Directorate General of Health Services (DGHS) of Bangladesh. Also, got the permission letter from Cox’s Bazar civil surgeon, Refugee Relief and Repatriation Commissioner (RRRC). Informed written consent was obtained from all participants according to the ‘Declaration of Helsinki’ regulation and guidelines. In case of child participants written consent was taken from the parents or legal guardians.

### Study population and sites

This is a prospective case-control investigation of acute respiratory infections (ARI) within the Forcefully Displaced Myanmar Nationals (FDMN). Participants were enrolled from March 2018 to March 2020 from a total of 6 camps and their respective health care units (HCU) situated in Cox’s Bazar, Bangladesh, sheltering the largest refugee camps in the world.

A total of 538 ARI cases were enrolled and between the age 6 months to 40 years and the inclusion criteria were these Rohingya patients manifesting at least one of the clinical signs and symptoms following WHO criteria: 1) cough or dyspnea, onset within the last 10 days; 2) history of fever in the last 7 days or current tympanic fever measured at ≥38.0°C; 3) tachypnea (In children between 2 months and <1 year of age: breathing rate > 50 breaths per minute; between 1 year and <5 years of age: breathing rate > 40 breaths per minute; between ≥5 years and <18 years of age: breathing rate > 30 breaths per minute; In adults ≥18 years of age: breathing rate > 20 breaths per minute 4) lower chest wall indrawing (expected in children ≤3 years old only) [1]. Cases that meet any of the following exclusion criteria were ineligible to enroll 1) wheezing suggestive of asthma; 2) a one-month (30-day) exclusion period following the date of discharge from the hospital; 3) ongoing or previously prescribed asthma or on corticosteroid treatment; and 4) recent intravenous antibiotic therapy.

In this study, we also enrolled a control group (n = 514) matched with the cases in terms of age, sex, and catchment area of Rohingya individuals aged ≥3 months without having any symptoms of acute or chronic infections including respiratory, COBP, digestive, and cutaneous.

Prior to enrolment, written informed consent was obtained from the participants, and in the case of minors, each child’s parents or legal guardians. In addition, demographic and clinical data were documented in case report forms (CRFs) at the time of specimen collection.

### Specimen collection

NPS and blood specimens were drawn from the ARI cases whereas only NPS samples from control group by trained personnel using standard operating procedures. Laboratory works of the collected samples were later conducted at the Institute for Developing Science and Health Initiatives (ideSHi), International Centre of Diarrhoeal Disease Research, Bangladesh (icddr,b) and Bangladesh Institute of Tropical and Infectious Diseases (BITID).

During NPS collection, a sterile cotton-flocked swab was soaked in saline and then placed approximately 1 to 1.5 cm into the nostrils and rotated against the anterior nasal mucosa for 3 seconds. Then the NPS sample was placed into universal transport media (UTM) (Copan Diagnostics Universal Transport Medium (UTM-RT) in Screw-Cap Tube)(DMEM, Gibco-BRL, Life Technologies, Paisley, Scotland; penicillin 10,000 U/ml, and streptomycin 10,000 IU/ml, BioWhittaker, MA; NaHCO3; HEPES buffer, 1M, Gibco; L-Glutamate 200 mM; Fungizone (Amphotericin B) 250 μg/ml; bovine serum albumin, Fraction V, 7.5%, Gibco) for detection of bacteria and viral pathogens. The collected specimens were immediately placed at 2–8°C and the samples were kept at -70°C for later identification of viral and bacterial pathogens following nucleic acid extraction. The extracted nucleic acids were then subjected to qRT-PCR. In addition, blood samples were collected only from the case group in EDTA tube for bacterial and viral pathogen detection by qRT-PCR, Aerobic Hemoculture bottle and Pediatric Aerobic Hemoculture bottle for blood culture as well as observation of antimicrobial resistance pattern.

### Culture of bacteria from blood specimens

Haemoculture assay was performed only for the case group using BacT/ALERT (bioMérieux, Marcy l’Etoile, France) automated blood culture systems. Blood cultures were performed for 464 out of 538 cases because of sample transportation issues. In children under-five, the collected blood volume was 1-3 mL, while in children aged five and over the blood volume was 6 mL. Besides, antimicrobial susceptibility testing was performed for all clinically relevant strains.

### Molecular biology testing of NPS and blood samples

Bacterial DNA was extracted from both blood and NPS samples using the QIAamp DNA Mini kit (Qiagen, Hilden, Germany), following the manufacturer’s instructions. A final elution volume of 50μL of DNA was stored in 1.5mL Eppendorf tubes at –20°C until amplification. The extracted DNA was then tested for the detection of three most common bacteria of pneumonia, including *S. pneumoniae*, *S. aureus* and *H. influenza type b* by real-time triplex PCR assay. This triplex PCR has been used in previous studies [24, 25]. Amplification was performed in a CFX96 Touch Real-Time PCR machine (Bio-Rad Laboratories, Inc). Positive and negative controls were included in each experiment. Those with a Ct value of 40 or less were identified as positive in the qRT-PCR test. A threshold value of 40 was used in each experimental run and we adjusted the RFU (relative fluorescence units) cut-off value according to the data validation team and values greater than the cut-off were considered positive. Additionally, *S. pneumoniae* serotyping was done with the multiplex RT-PCR described previously for 40 serotypes [26]. For eight viruses (RSV, HMPV, HPIV-1, HPIV-2, HPIV-3, INF-A, INF-B and ADV) detection only from the NPS samples of a total of 1000 samples (512 cases and 488 controls) where nucleic acids were extracted using Nucleic Acid Extraction kit (Chemagic viral NA/gDNA kit (PerkinElmer) and the extraction Machine: chemagic 360 instrument. qRT-PCR was done with iTaq Universal Probes One-Step Kit (Bio-Rad) and the machine used was PCR Machine: CFX96 Touch Real-time PCR Detection System (Bio-Rad). The primers and probes used for virus detection, Table 1.

**Table 1:** Primer and Probe sequences used for eight viruses detection by qRT-PCR following nucleic acid extraction.

Primer’s name	Nucleotide Sequence 5’-3’
RSV Forward	GGC AAA TAT GGA AAC ATA CGT GAA
RSV reverse	TCT TTT TCT AGG ACA TTG TAY TGA ACA G
RSV probe	6 FAM-CTG TGT ATG TGG AGC CTT CGT GAA GCT-BHQ1
HMPV Forward	CAA GTG TGA CAT TGC TGA YCT RAA
HMPV Reverse	ACT GCC GCA CAA CAT TTA GRA A
HMPV probe	6 FAM-TGG CYG TYA GCT TCA GTC AAT TCA ACA GA-BHQ1
HPIV1 Forward	ACA AGT TGT CAA YGT CTT AAT TCR TAT
HPIV1 Reverse	TCG GCA CCT AAG TAR TTY TGA GTT
HPIV1 Probe	6 FAM-ATA GGC CAA AGA “T”TG TTG TCG AGA CTA TTC CAA
HPIV2 Forward	GCA TTT CCA ATC TAC AGG ACT ATG A
HPIV2 Reverse	ACC TCC TGG TAT AGC AGT GAC TGA AC
HPIV2 Probe	6 FAM-CCA TTT ACC “T”AA GTG ATG GAA TCA ATC GCA AA
HPIV3 Forward	TGG YTC AAT CTC AAC AAC AAG ATT TAA G
HPIV3 Reverse	TAC CCG AGA AAT ATT ATT TTG CC
HPIV3 Probe	6 FAM-CCC RTC TG“T” TGG ACC AGG GAT ATA CTA CAA A
Inf B Forward	TCC TCA AYT CAC TCT TCG AGC G
Inf B Reverse	CGG TGC TCT TGA CCA AAT TGG
Inf B Probe	FAM-CCA ATT CGA GCA GCT GAA ACT GCG GTG- BHQ1
Inf A Forward	GAC CRA TCC TGT CAC CTC TGA C
Inf A Reverse	AGG GCA TTY TGG ACA AAK CGT CTA
Inf A Probe	FAM-TGC AGT CCT CGC TCA CTG GGC ACG-BHQ1
Adeno Forward	GCC CCA GTG GTC TTA CAT GCA CAT C
Adeno Reverse	GCC ACG GTG GGG TTT CTA AAC TT
Adeno Probe	6 FAM-TG CAC CAG ACC CGG GCT CAG GTA CTC CGA-BHQ1

Moreover, whole blood samples were collected from only cases (in EDTA tube) extracted using QIAamp DNA blood mini kit (Qiagen, Hilden, Germany), and quantitative Triplex PCR assay was conducted on 5 μL of extracted DNA to identify *S. pneumoniae*, *Staphylococcus aureus* and *H. influenzae b* following the published protocols [24, 25].

### Comparison of Ct values

Ct values for ARI pathogens were compared as Ct value indicates the first PCR cycle in which the fluorescent signal for the target (DNA/RNA) is detected higher than the detection threshold. The quantity of target is inversely proportional to its Ct value which provides a semi-quantitative evaluation of viral/bacterial load where a low Ct value indicates a high viral load and the other way around [27, 28].

### Statistical analysis

Descriptive statistics were utilized to define the study participants based on several characteristics, and the frequency of each characteristic was reported with percentage. For the comparison of characteristics and pathogen detection, the chi-square test was used. In addition, in both the case and control groups, two-tailed chi-square was used to quantify the prevalence of the detected pathogen and co-infection frequencies in different age groups. Also, a Pearson correlation analysis was conducted to determine the pathogens’ correlation pattern. Each virus was fitted with a logistic regression model. The significance of associated factors was determined using an odds ratio with a 95% confidence interval. A level of significance was defined as a P value of less than 0.05. For statistical analysis, we used IBM SPSS Statistics for Windows (Version 20.0. Armonk, NY: IBM Corp) and STATA (Version SE-15, StatCorp LLC, College Station, TX) and R (version 4.1.2). Microsoft Access (Microsoft Office Professional 2013) was also used for data entry.

## Results

### Demographic and clinical characteristics

Prior to enrollment, participants were examined for clinical signs and symptoms by physicians for ARI case definitions. A total of 538 cases were enrolled where 54% (291) were male. Among the age groups, 59% (320) were ≤5 years of age and the rest were older. A set of age and sex-matched control participants (n = 514) were also enrolled, Table 2.

**Table 2:** Demographic characteristics of ARI cases and control group.

	ARI Cases (n = 538)	Controls (n = 514)
**Sex**		
**Male**	291 (54)	254 (49)
**Female**	247 (46)	260 (51)
**Age**		
**≤5 years**	320 (59)	299 (58)
**>5 years**	218 (41)	215 (42)

Among the enrolled cases, cough was the most prevalent symptom, specifically dry cough and around 97% were classified as pneumonia cases according to WHO case definition criteria [1]. Control group came to the health care unit seeking medical help and other reasons specifically for external injury, back pain or joint diseases, immunization, and urinary diseases. ARI cases were enrolled with different clinical symptoms, where it was highest with dry cough. Other predominant symptoms were dyspnea, tachypnea and lower chest wall in drawing. Most of the clinical symptoms were associated with bacterial and viral infection as well as co-infection between bacteria-bacteria and bacteria-virus where no significant etiological differences were observed for clinical symptoms, Table 3.

**Table 3:** Clinical characteristics and pathogen detection distribution in ARI case group.

Symptoms for ARI cases	ARI Cases (n/%)	Single infection		Co-infection		
		Bacteria (n/%)	Virus (n/%)	Bacteria-Bacteria (n/%)	Virus-Virus (n/%)	Bacteria-Virus (n/%)
**Cough**	538(100)	459(85)	158(30)	126(24)	6(1)	138(26)
**Productive**	52(9)	46(10)	11(6)	16(12)	0	11(7)
**Dry**	486(90)	413(89)	147(93)	110(87)	6(100)	127(92)
**Dyspnea**	534(99)	455(85)	157(30)	123(24)	6(1)	137(26)
**Temperature**						
**≥ 102.2**	20(3)	18(3)	8(5)	6(4)	1(16)	7(5)
**<102.2**	518(96)	441(96)	150(94)	120(95)	5(83)	131(94)
**Wheezing**	0	0	0	0	0	0
**Hospitalized** *	0	0	0	0	0	0
**Prior antibiotic therapy**	0	0	0	0	0	0
**Tachypnea**						
**<1 year (>50)**	17(94)	16(94)	5(83)	3(75)	2(100)	5(83)
**1 to 5 years (>40)**	229(87)	206(86)	76(86)	57(90)	3(75)	71(87)
**>5 year (>30)**	215(89)	169(88)	52(85)	51(87)	0	41(82)
**Lower chest in drawing (<3 years of age)**	135(25)	126(93)	51(40)	30(24)	4(3.20)	47(37)
**Oxygen saturation**						
**<95**	15(2)	14(3)	3(1)	5(3)	0	3(2)
**≥95**	523(97)	445(96)	155(98)	121(96)	6(100)	135(97)

*hospitalized represents data from the OPD-based hospitalization in the study sites, tertiary hospitalization data not available

### Overall pathogen detection in ARI cases and controls group

In summary, comparable proportion of bacterial pathogens was found among cases 85% (459/538) and controls 87% (452/514) and was not significantly different for at least one of the three bacteria tested. Similar trend was found in gender and age groups between cases and controls. However, children of ≤5 years were at higher risk of bacterial pathogen detection than those >5 years of age both for cases 90% (289/320) vs 77% (170/218), p<0.05 and controls 93% (280/299) vs 80% (172/215), p<0.05. For viral infections, 512 cases and 488 control specimens were tested (for eight viral pathogens, had at least one positive) where higher detection rate was also found in cases than controls 30% (158/512) vs 16% (80/488), p<0.05. Children ≤5 years of age were the high-risk group than those >5 years of age group both in cases 34% (104/301) vs 17% (51/284), p<0.05 and controls 26% (54/211) vs 15% (29/204), p<0.05. Viral detection rate was higher in the case group in both male and female groups compared to that seen in the males and females in the control group. While observing co-infection (any two different pathogens detected among the 11 pathogens tested), the highest rate was found in the control group than in cases (37% vs 26%), p<0.05. Among the age groups, those ≤5 years of age were found to have higher detection rates than those >5 years of age in case 31% (96/301) vs 19% (42/211), p<0.05 but in control group, it was comparable, Table 4.

**Table 4:** Positivity of bacterial and viral pathogens and their co-infection rate among the ARI cases and control group.

	All bacteria tested		All viruses tested			All bacteria and viruses tested ^a^		
	ARI case (n = 538)	Control (n = 514)	ARI case (n = 512)	Control (n = 488)		ARI case (n = 512)	Control (n = 488)	
**All**	459(85.32)	452(87.94)	158(30.86)	80(16.39)	*	138(26.95)	181(37.09)	*
**Sex**								
**Male**	253(86.94)	225(88.58)	86(30.82)	39(16.18)	*	76(27.24)	94(39.00)	*
**Female**	206(83.40)	227(87.31)	72(30.90)	41(16.60)	*	62(26.61)	87(35.22)	*
**Age group**								
**≤5 years**	289(90.31)	280(93.65)	104(34.55)	51(17.96)	*	96(31.89)	103(36.27)	
**>5 years**	170(77.98)	172(80)	54(25.59)	29(14.22)	*	42(19.91)	78(38.24)	*
	*	*	*			*		

Number and proportion of at least one positive bacterial/viral pathogen shown in the table. Three bacterial and eight viral pathogens were tested.

^a^Co-detection was defined as detected by at least two bacterial or viral pathogens including three bacterial and eight viral pathogens.

* indicates significant p<0.05

### Positive spectrum of viruses and bacteria among cases and control groups detected from NPS specimens by qRT-PCR

**Fig 1:**
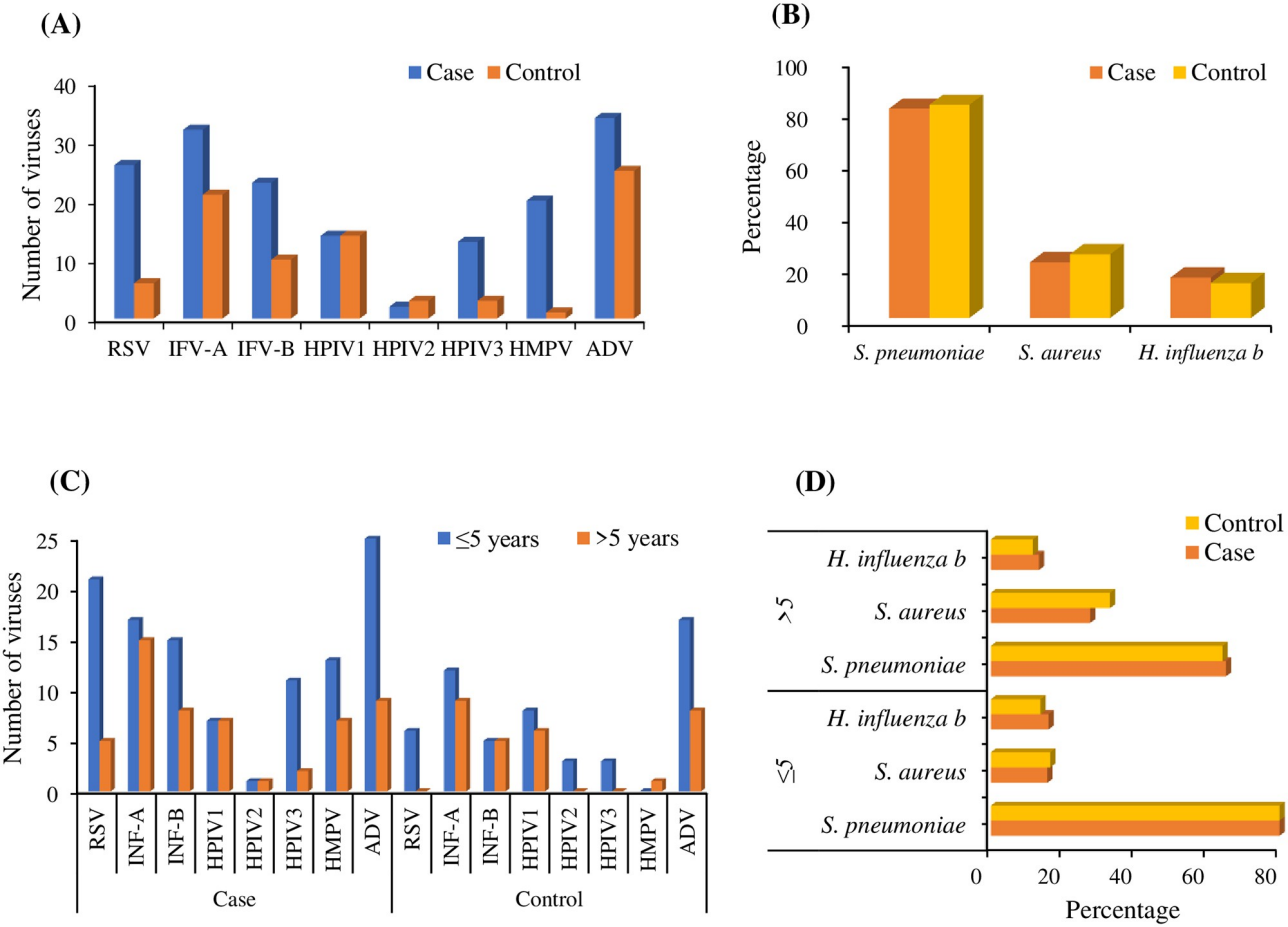
Pathogen detection from NPS samples by qRT-PCR in cases and control group. (A) Number of eight viruses detected in cases and control (B) Percentage of major three bacteria detected in cases and control group (C) Age-specific distribution number of eight viruses in cases and control group (D) Age-specific percentage of three major bacteria in cases and control group.

Among the bacteria detected, *S. pneumoniae* was the most frequently detected bacterial pathogen both in case 80% (414/512) and control groups 82% (402/488) followed by *S. aureus* 21% (110/512) vs 24%(120/488) and *H. influenza* b (15% vs 13%). Moreover, high detection rate was observed for ≤5 years of age than >5 years of age group both in cases and control group for *S. pneumoniae* 85% (272/320) vs 65% (142/218), p<0.05 for cases and 88% (264/299) vs 64% (138/215), p<0.05 for controL, and *H. influenza b* (15% vs 13% for cases and 13% vs 11% for controls) where detection was found higher in control group for *S. aureus* 15% vs 27%, p<0.05 for cases and 16% vs 33%, p<0.05 for controls. For viruses, the overall detection number was higher in the case group than control group for all eight viruses tested by qRT-PCR from the NPS samples. In cases, number of detection was highest for ADV (34) followed by IFV-A (32), RSV (26), IFV-B (23), and HMPV (20). Within the genotypes of HPIV, the highest number was observed in HPIV1 (14), HPIV3 (13), and HPIV2 (2). For controls, the sequence of detection numbers for the viruses was ADV >IFV > HPIV and RSV. Some viruses were found significantly higher (p<0.05) for ≤5 years age group in cases than control like RSV (21 vs 6, p<0.05), IFV-B (15 vs 5, p<0.05), HPIV-3 (11 vs 3, p<0.05) and HMPV (13 vs 0, p<0.05). Also, ≤5 years age group was detected with highest number of viruses both in cases and control group. In case group, it was observed like ADV (25 vs 9), RSV (21 vs 5,p<0.05), IFV-a (17 vs 15), INF-B (15 vs 5), HMPV (13 vs 7), HPIV-3 (11 vs 2), HPIV-1 (7 vs 7) and HPIV-2 (1 vs 1). Similarly, in control group ≤5 years age group was also found with higher detection than >5 years age such as IFV-A (15 vs 9), IFV-B (8 vs 5), ADV (9 vs 8), HMPV (7 vs 1) and RSV (6 vs 0, p<0.05). Both in case and control group RSV was found significantly higher for ≤5 years age group. Within genotypes of HPIV number of detection were HPIV-1 (7 vs 6), HPIV-2 (1 vs 0), HPIV-2 (2 vs 0). Overall, ≤5 years age group was found at high risk both for case and control than the >5 years age group (Fig 1).

### Single and co-infection in cases and controls

**Fig 2:**
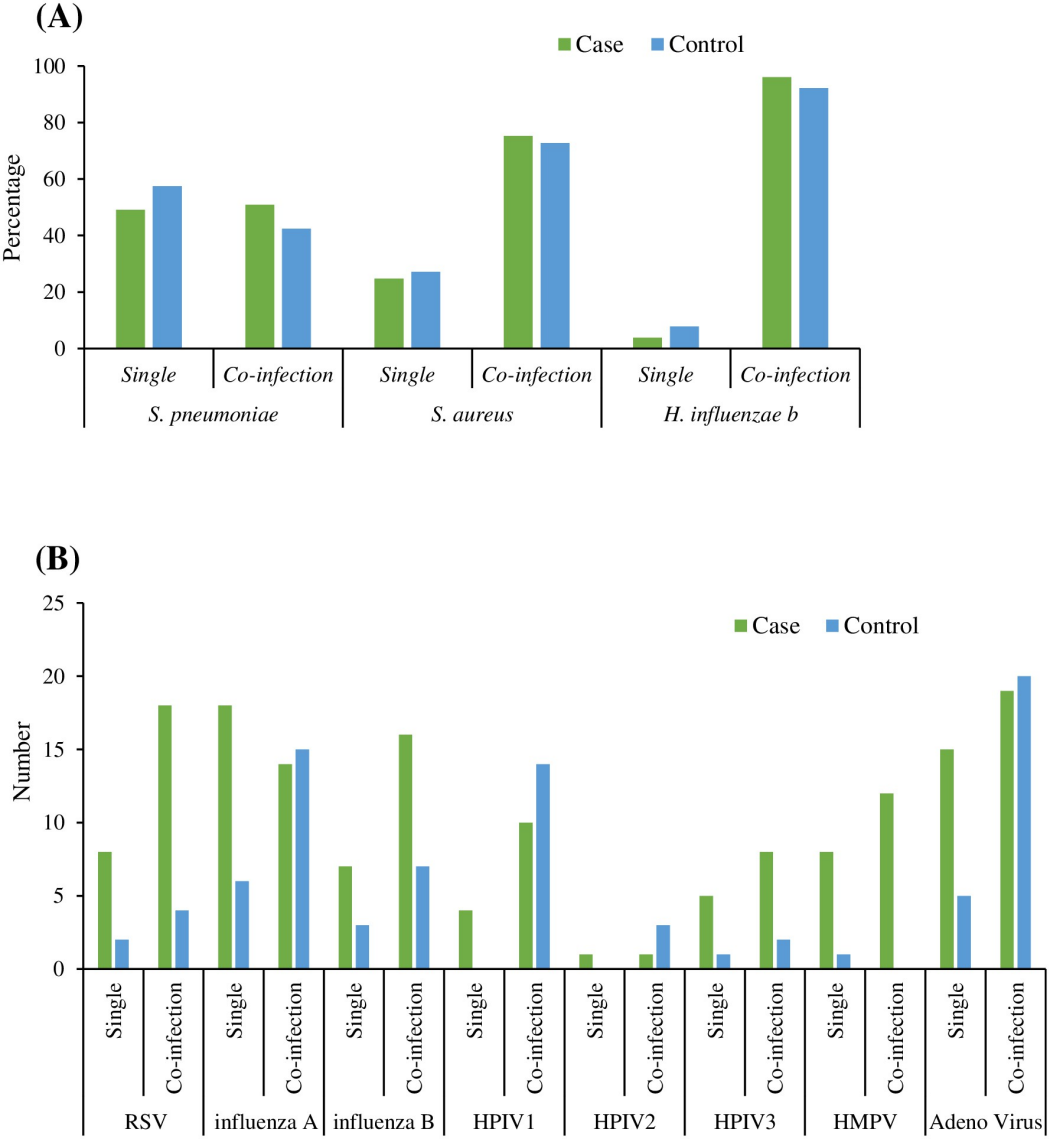
Single and co-infection frequencies of each pathogen identified from the NPS samples among cases and control group. Bacterial co-infection was calculated as any two bacteria out of three found positive simultaneously. Viral co-infection was defined as any two viruses out of eight detected positive simultaneously. (A) Single and co-infection percentages of bacteria detected in cases and control detected in NPS samples by qRT-PCR. (B) single and co-infection percentages of viruses detected in cases and control group in NPS samples by qRT-PCR.

Single infection frequency was found similar between the case and control groups as well as the co-infection frequency considering three bacteria tested. However, particularly for *S. pneumoniae*, no significant difference was found between single and co-infection both in cases and also in control group. However, single infection was significantly high in control group (p<0.05) and co-infection was high in case group. In addition, co-infection was found significantly higher than single infection (p<0.05) both in case and control groups for both *S. aureus and H. influenza b*. In the case of co-infection among viruses, co-infection was found to be higher than single infection for all the viruses except IFV-A in the case group. Similar patterns were observed for control group for all the viruses except for HMPV. However, single infection as well as co-infection numbers were higher in cases than control group for RSV, IFV-B, HPIV-3 and HMPV. In addition, control group was found in high viral positive number over case group for co-infection (IFV-A, HPIV1, ADV) and no single infection was found higher for the control group over the cases (Fig 2).

### Correlation pattern between bacterial and viral pathogens

**Fig 3:**
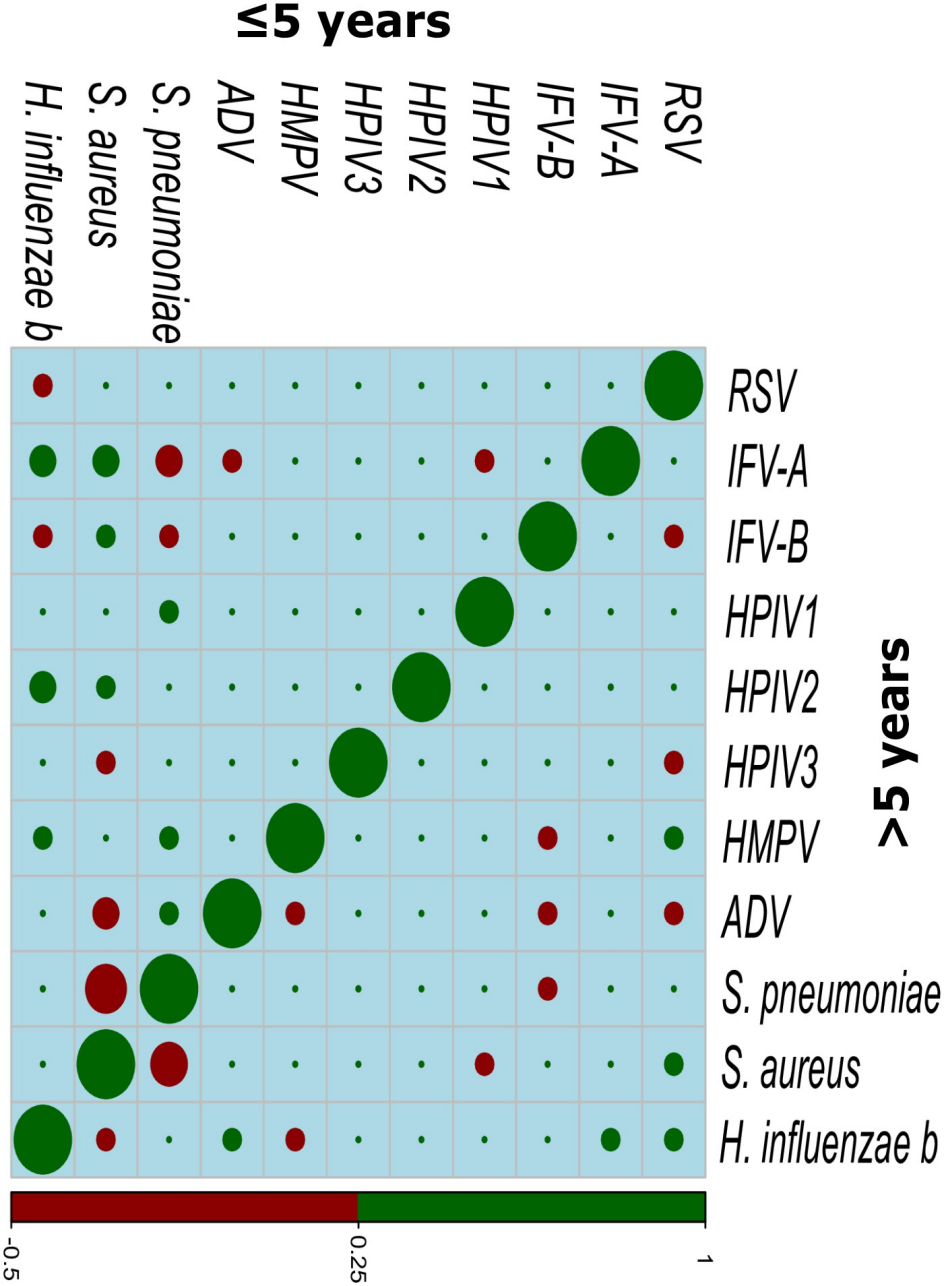
Correlation pattern between different pathogens. Correlation patterns were observed between each pathogen for three bacteria and eight viruses tested from NPS samples by qRT-PCR in the case group for both ≤5 age and >5 age.

Most predominant correlations were found between bacteria and viruses where the patterns differed in age groups. In ≤5 age group most predominant were observed for *H. influenza b* with RSV, IFV-A with ADV; RSV with *S. aureus* and HMPV where they were positively correlated. In contrast, no positive virus-virus correlation was found except for RSV with HMPV where negative viral-viral correlation was found for RSV with ADV, HPIV3 and IFV-B; ADV with IFV-B, HMPV, HMPV and IFV-B. Moreover, *H. influenza b*, *S. aureus* and *S. pneumoniae* were negatively correlated with each other. Also, *H. influenza b* and *S. aureus* were positively correlated with IFVs where *S. pneumoniae* was found negatively correlated with IFVs and less likely to coexist with them found particularly for ≤5 age group On the other hand, in >5 age the positive correlation patterns were found *S. aureus* with IFV-A, IFV-B and HPIV2 and *H. influenza b* with IFV-A, HMPV and HPIV2 and *S. pneumoniae* with HPIV1, HMPV and ADV. However, in both age groups no bacteria-bacteria positive correlation was observed where negative bacteria-bacteria correlation was found for *S. pneumoniae* with *S. aureus* in both age groups (Fig 3). However, such correlation analysis was not possible for all pathogens in control group as correlation did not exist for some pathogens in this group.

Moreover, logistic regression analysis data showed the prevalence of each pathogen’s distribution frequency between case and control and their age-specific distribution. From the analysis, no significant difference or association was observed for three major bacteria tested. However, for viruses, among the respondents in cases were about 4 times more likely to be RSV positive than the controls. Similarly, for influenza B it was about 2 times, for HPIV3 it was 4 times and for HMPV it was significantly higher about 20 times whereas no significant associations were observed for other viruses. While considering the age group analysis in cases and control, respondents aged >5 years were 75% less likely to be RSV positive than those aged ≤5 years. Also, HPIV3 80% less likely to be RSV for >5 years age, ADV 45% less likely to be for >5 years age where significant associations were observed Table 5.

**Table 5:** Associated factors for viruses tested for cases (n = 512) and control group (n = 488).

Variables	RSV	IFV-A	IFV-B	HPIV1	HPIV2	HPIV3	HMPV	ADV
	OR (95% CI)	OR (95% CI)	OR (95% CI)	OR (95% CI)	OR (95% CI)	OR (95% CI)	OR (95% CI)	OR (95% CI)
**Control**	1	1	1	1	1	1	1	1
**Case**	4.32(1.76-10.61)*	1.48(0.84-2.61)	2.25(1.06-477)*	0.95(0.45-2.02)	0.63(0.10-3.79)	4.21(1.19-14.90)*	19.78(2.65-147.99)*	1.31(0.77-2.24)
**Age**								
**≤5 years**	1	1	1	1	1	1	1	1
**>5 years**	0.25(0.10-0.66)*	1.18(0.68-2.06)	0.92(0.45-1.87)	1.23(0.58-2.61)	0.35(0.04-3.14)	0.20(0.04-0.88)*	0.87(0.36-2.14)	0.55(0.31-0.99)*

Note:

* = p<0.05, OR = Odds Ratio, CI = Confidence Interval

Dependent variables are the eight viruses and each of them are dichotomous variables. Gender-specific analysis was skewed.

### Semi-quantitative bacterial and viral load in cases and control group

**Fig 4:**
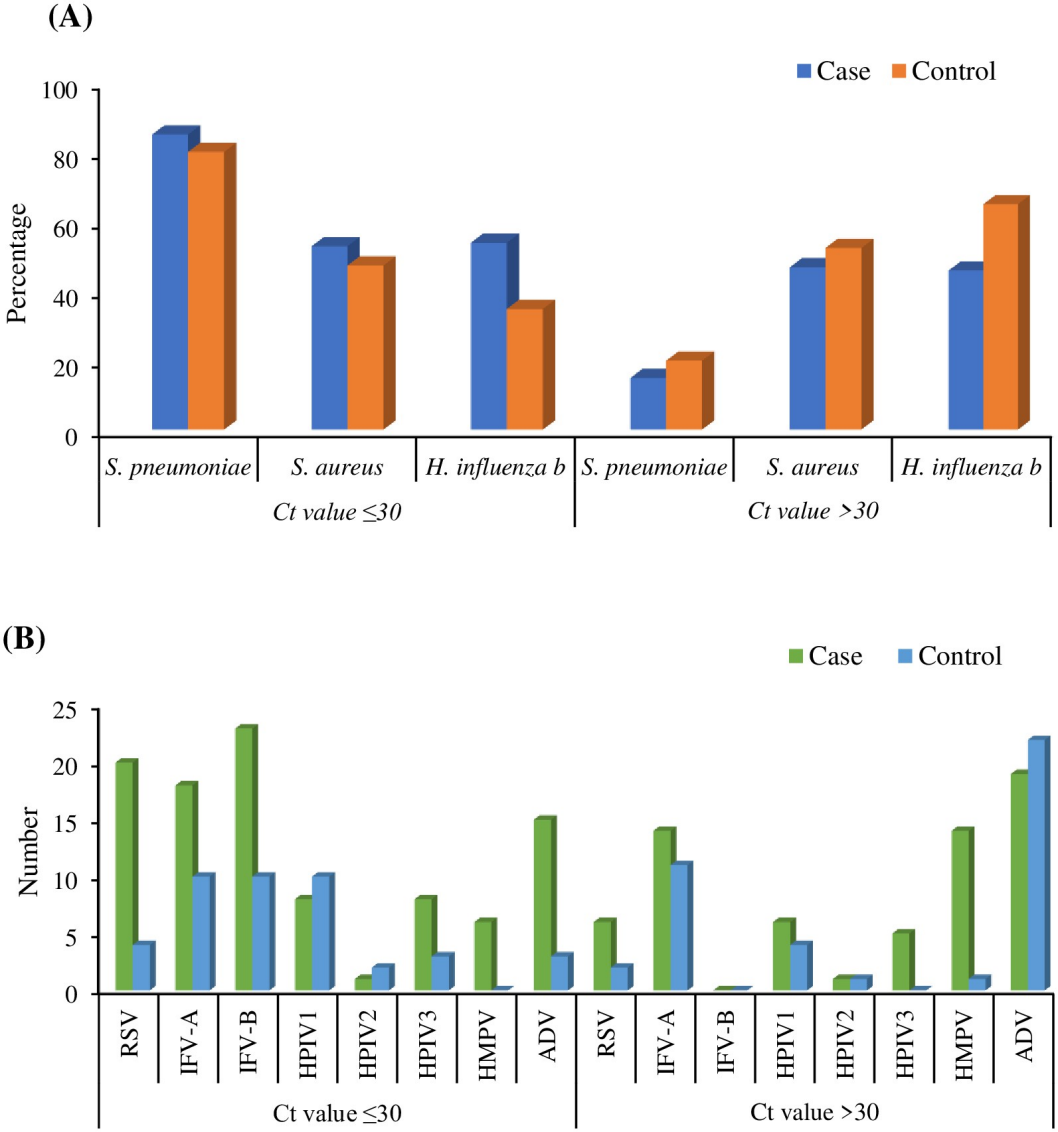
Bacterial and viral semi-quantitative load. (A) Bacterial load detection in NPS samples by qRT-PCR calculated according to Ct values in the case and control group. (B) Viral load detection in NPS samples by qRT-PCR was calculated according to Ct values in the case and control groups. Higher bacterial/viral load considered for Ct≤30.

The most frequent pathogen was *S. pneumoniae*, detected by qRT-PCR both in cases and in controls, followed by *S. aureus* and *H. influenza b* from the NPS samples tested. However, their Ct value was calculated to measure the semi-quantitative bacterial load. The value of the qRT-PCR cycle threshold (Ct) indicates the first PCR cycle in which the fluorescent signal for the target (DNA/RNA) is detected is higher than the detection threshold. The quantity of target is inversely proportional to its Ct value which provides a semi-quantitative evaluation of viral/bacterial load so that a low Ct value indicates a high viral load and the other way around. Interestingly, while considering the semi-quantitative bacterial load according to Ct values targeting ≤30 the detection percentage was found higher in cases than in control and high Ct value indicating lower bacterial load (Ct>30) was found predominant in control group. For viruses, a low Ct value indicating higher viral load category was seen in the case group than the control group for all the viruses except for HPIV-1, 2, and ADV (Fig 4).

### Pneumococcal serotypes/serogroups distribution in ARI cases and control group

**Fig 5:**
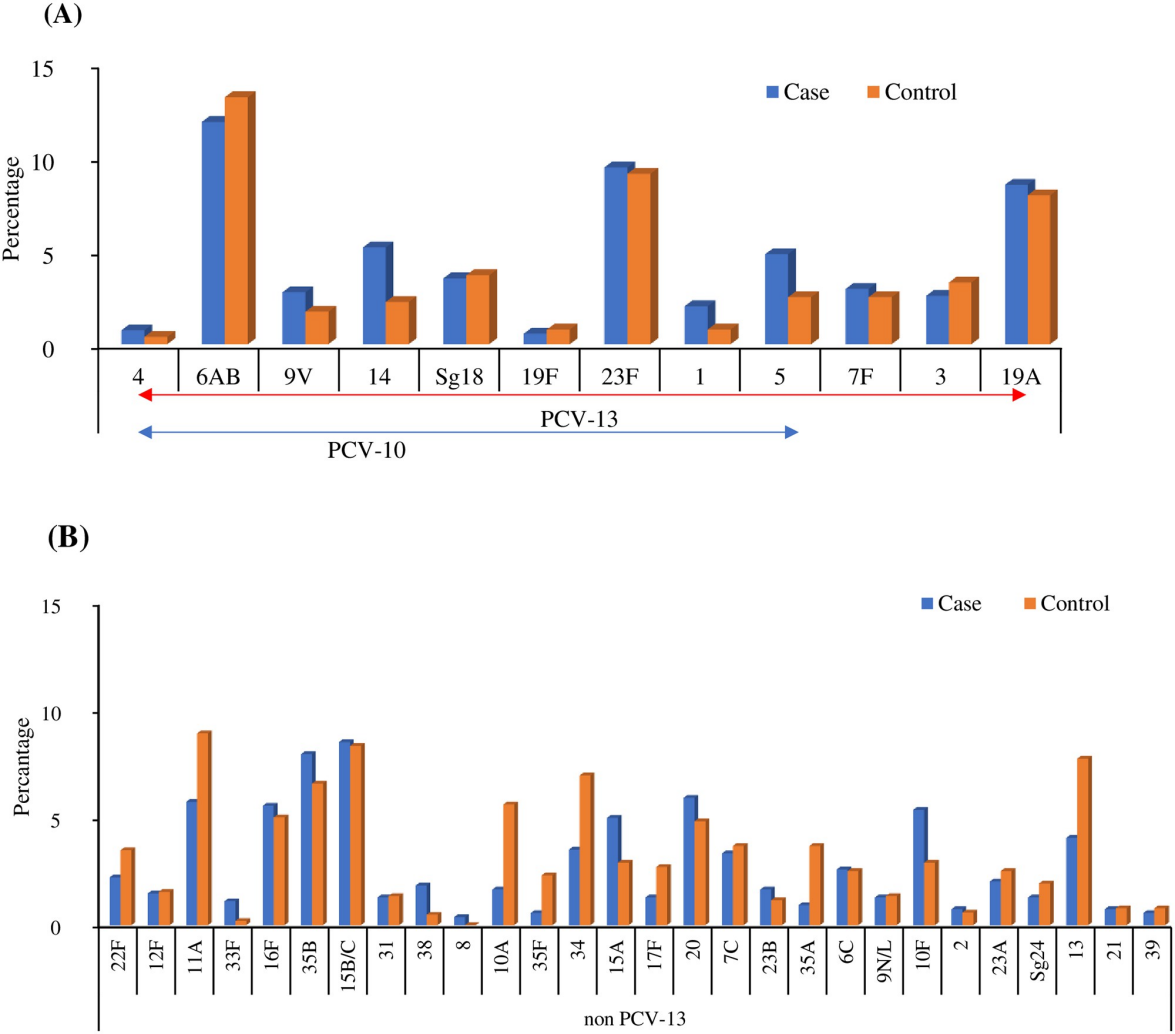
*S. pneumoniae* serotype distribution in case and control. (A) *S. pneumoniae* serotypes distribution percentage in cases and control where serotypes were covered within PCV-10 (pneumococcal conjugate vaccine 10) and PCV-13 (pneumococcal conjugate vaccine 13) detected from NPS samples by qRT-PCR (B) *S. pneumoniae* serotypes distribution percentage in cases and control where serotypes were covered other than PCV-13 detected from NPS samples by qRT-PCR.

In the FDMN population, pneumococcal conjugate vaccines (PCV) were introduced to 41% of ARI cases and 45% of control group in our study population which includes both PCV-10 and PCV-13. Serotypes of *S.pneumoniae* determined showed that 30.6% (221) belong to PCV10, 41.1% (297) and 60% (425) for PCV13 and non-PCV13 respectively. Among the 722 numbers of isolates, serotypes/serogroups including 221 (52%) were found under PCV10 serotypes/serogroups and 297 (41%) were non-PCV13 serotypes/serogroups. There was no significant difference found between cases and controls when considering multiple serotypes/serogroups. However, some serotypes were found in higher percentages in cases and controls such as for PCV-13, e.g. 6AB, 23F and 19A. In non-PCV13 serogroups 22F, 11A, 10A, 35F, 34, 17F, 35A and 13 serotypes were found in higher numbers in the control group than in the case group where 35B, 15A, 20, and 10F serotypes were found in higher numbers in the case group. The most predominant serotypes were 6AB (11.9%, 64/538) in case and 13.2%, 68/514) in control followed by 23F in and 19A was found as the most predominant both in case and control. In non-PCV13 category highest proportion of serotypes are as follows 15B/C, 35B, 16F, 20, 11A, 15A, 10F and 13 for cases where in control 11A, 15B/C, 13, 34, 35B, 10A, 16F, 20, 22F, 7C and 35A (at least 4% was selected as high proportion). It was seen that 402 isolates were positive for lytA out of 414 (97.02%) in case and 94.27% in controls, indicating the presence of non-encapsulated non-typeable *S. pneumoniae* (Fig 5).

However, no significant differences were found when considering the presence of multiple serotypes between cases and control group, Table 6.

**Table 6:** Multiple serotypes in cases and control group.

Number of multiple Serotypes	Case group	Control group
**2**	115	105
**3**	57	49
**4**	25	22
**5**	9	14
**6**	2	5
**7**	1	2
**8**	0	1

**Fig 6:**
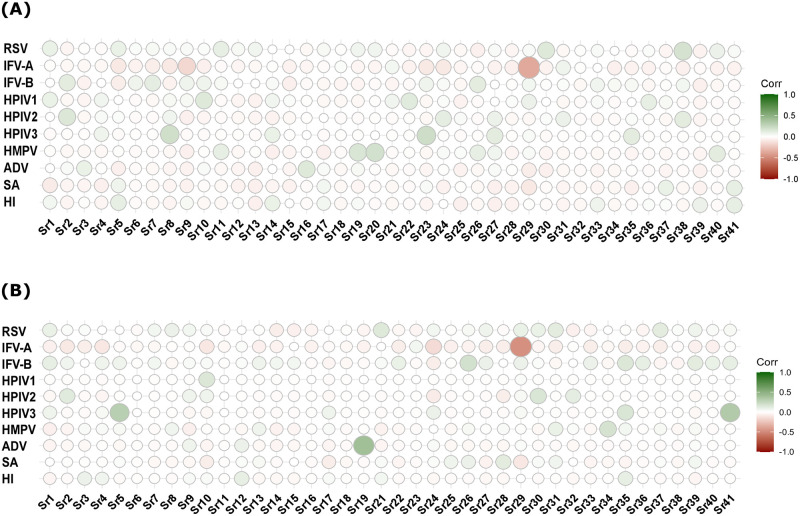
Correlation pattern between difference pathogens with *S. pneumoniae* serotypes. (A) Correlation between *S. pneumoniae* serotypes and other pathogens in the ARI case group were detected from NPS samples by qRT-PCR. (B) Correlation between *S. pneumoniae* serotypes and other pathogens in control group detected from NPS samples by qRT-PCR. Here, Sr1 = 19A, Sr2 = 22F, Sr3 = 3, Sr4 = 6AB, Sr5 = 14, Sr6 = 4, Sr7 = 12F, Sr8 = 9V, Sr9 = 23F, Sr10 = 11A, Sr11 = 33F, Sr12 = 7F, Sr13 = 16F, Sr14 = 35B, Sr15 = Sg18, Sr16 = 19F, Sr17 = 15B/C, Sr18 = 31, Sr19 = 38, Sr20 = 8, Sr21 = 10A, Sr22 = 35F, Sr23 = 1, Sr24 = 34, Sr25 = 15A, Sr26 = 17F, Sr27 = 20, Sr28 = 7C, Sr29 = lytA, Sr30 = 5, Sr31 = 23B, Sr32 = 35A, Sr33 = 6C, Sr34 = 9N/L, Sr35 = 10F, Sr36 = 2, Sr37 = 23A, Sr38 = Sg24, Sr39 = 13, Sr40 = 21, Sr41 = 39. Also, SA = *S. aureus*, HI = *H. influenza b*.

In addition, we wanted to see the correlation pattern between serotypes and other bacterial and viral pathogens tested for ARI cases. Correlation data showed that almost all the serotypes are correlated with other pathogens with positive and/or negative interactions. However, *S. aureus* showed less positive interactions among the tested pathogens, where it has positive interactions with Sr21 and Sr31. In addition, a high number of negative interactions were found for many serotypes such as 3, 4, 12F, 23F, 7F, 16F, Sg18, 19F, 31, 35F, 15A, 7C, 5, 35A, 9N/L, 2, and 13. In contrast, strong positive interactions with other pathogens were observed for several serotypes like 19A, 22F, 9V, 23F, 38, 8, 1, 17F, 20, Sg24 and 39. Where in control group, *S. aureus* showed a high negative interaction in a similar manner. In this group, a high number of negative interactions were found for many serotypes such as 4, 33F, 35B, Sg18, 19F, 31, 1, 15A, 7C, 6C, Sg24, 9N/L, and 2 where strong positive interactions were found for a few serotypes like 14, 11A, 38, 10F and 39 (Fig 6).

### Pathogen detection from blood specimens and antimicrobial resistance pattern

**Fig 7:**
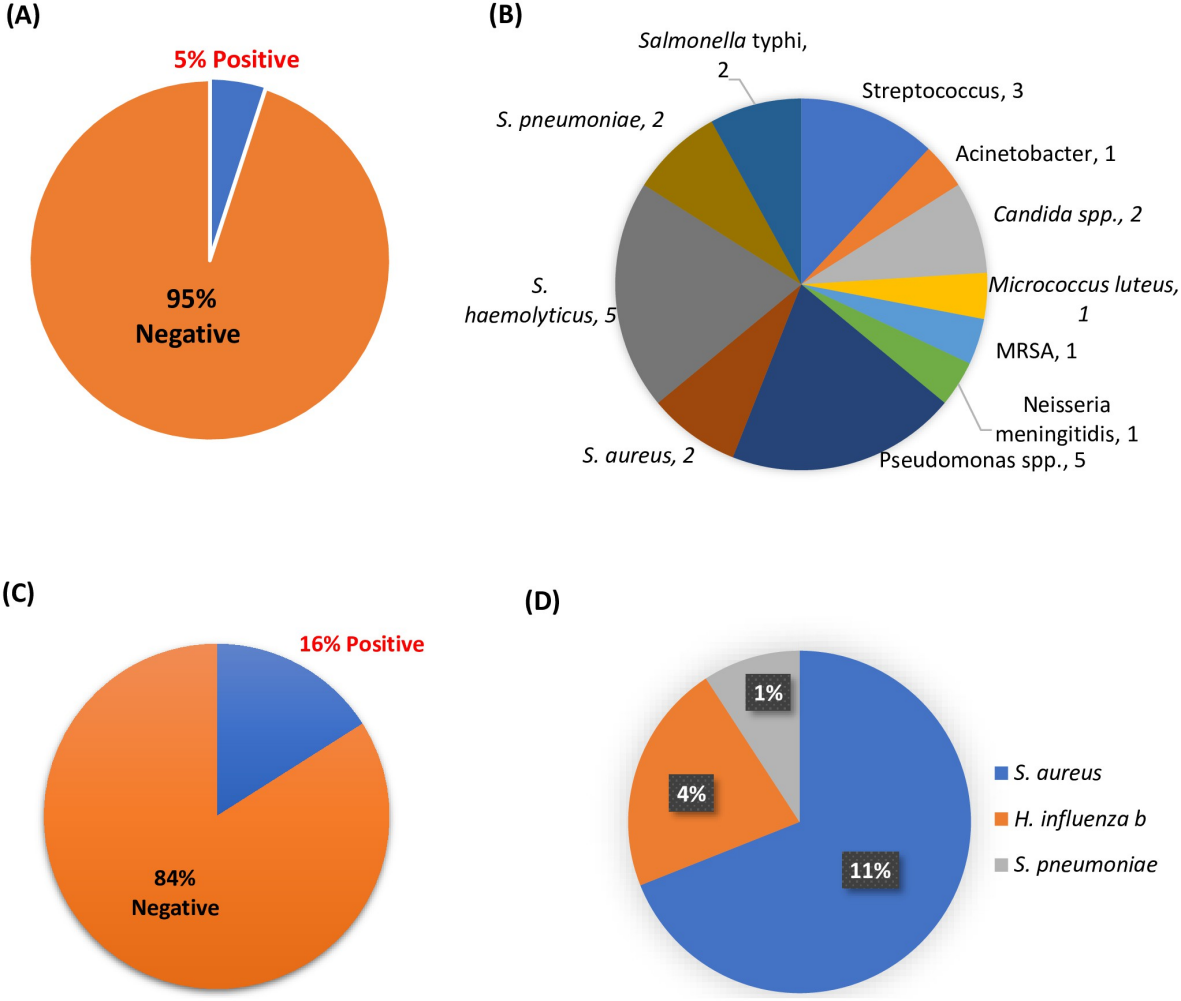
Pathogen identification from blood culture.

From the blood culture, only 5% tested positive for bacterial pathogens where *S. haemolyticus* (5) and *Pseudomonas spp* (5) was found in the highest number and only 2 *S. pneumoniae* were found (Fig 7).

**Fig 8:**
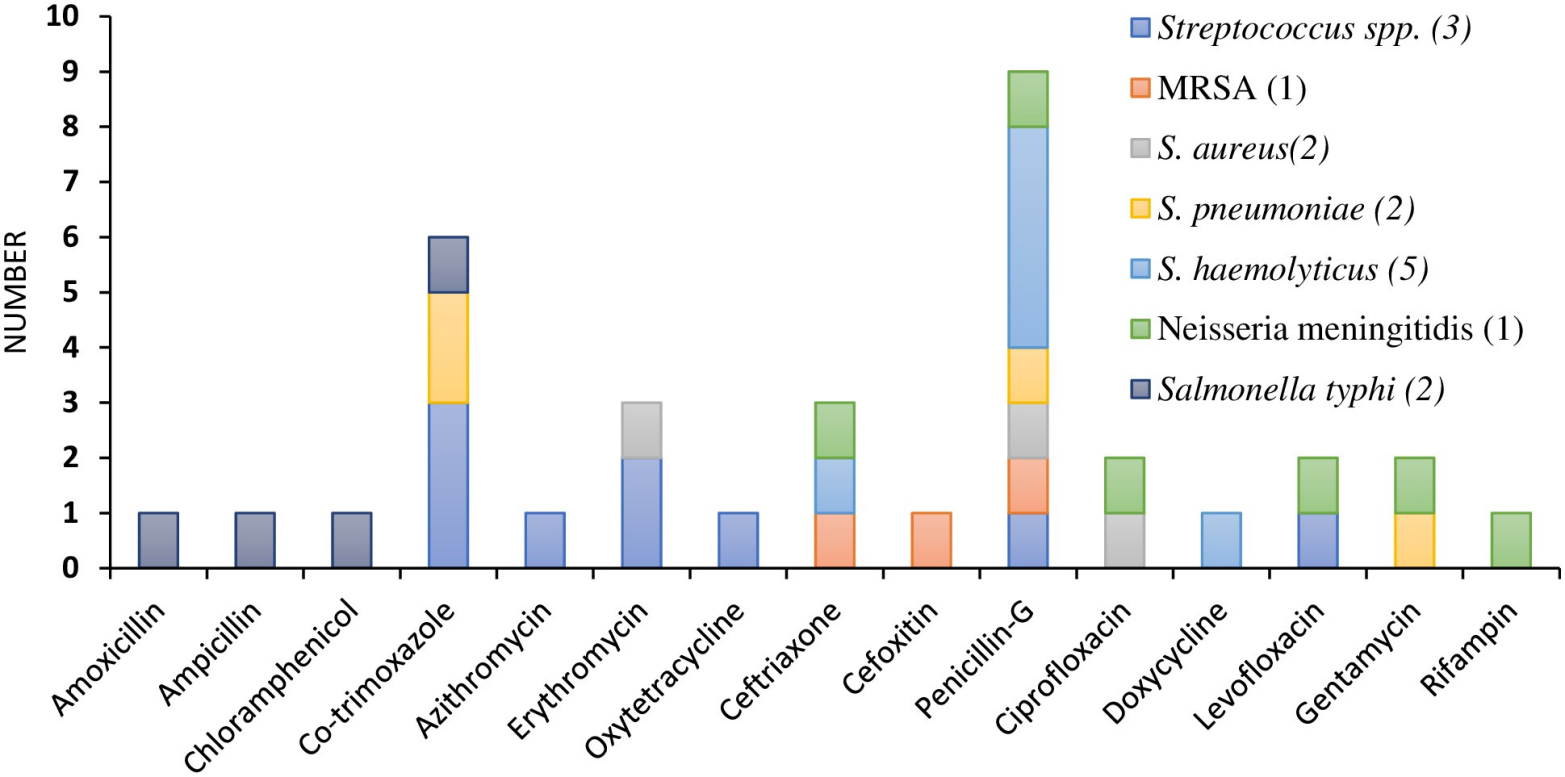
Antimicrobial resistance patterns for pathogen identification from blood culture.

In addition, antimicrobial resistance patterns showed isolated bacteria were most resistant to penicillin-G and cotrimoxazole and *S. pneumoniae* isolates were resistant to cotrimoxazole, penicillin-G, and gentamycin (Fig 8).

### ARI cases enrollment and pathogen detection frequency

**Fig 9:**
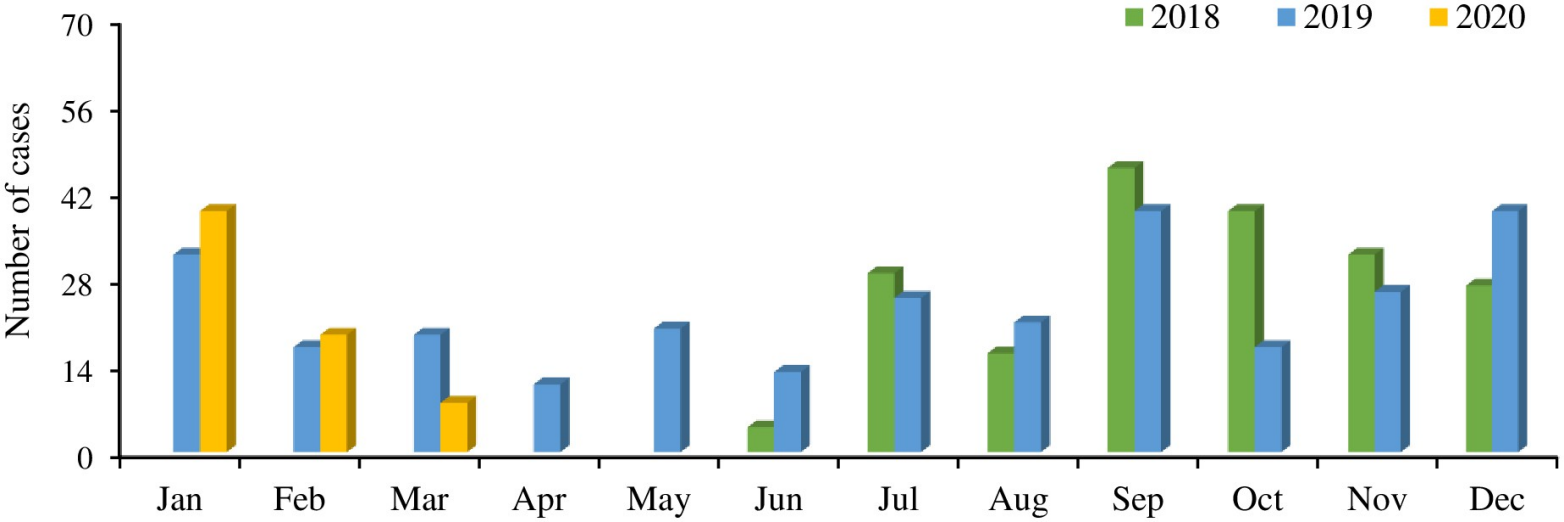
Month-wise case enrollment throughout the study period.

A total of 538 cases were enrolled from July 2018 to March 2020 in the study period. The rate of ARI case enrollment was highest during September then the detection rate was low and remained almost similar until January. In 2018, September (n = 46) was the month of highest enrollment, then it was gradually declining for ARI case enrollment till December (n = 27). Similarly, in 2019, the rate of cases enrollment peaked in September (n = 39), and fell sharply in October (n = 17) and after October there was a continuous upsurge in case enrollment which remained high till January (n = 32). Again, after January, the case enrollment dropped gradually and remained low till June (n = 13). A similar trend was also observed in 2020 where a sharp peak was found in January (n = 39) and then a sharp decline till March (n = 8). In summary, most of the cases’ enrollment (80%) was between July and January (Fig 9).

#### Seasonal distribution of ARI pathogens

**Fig 10:**
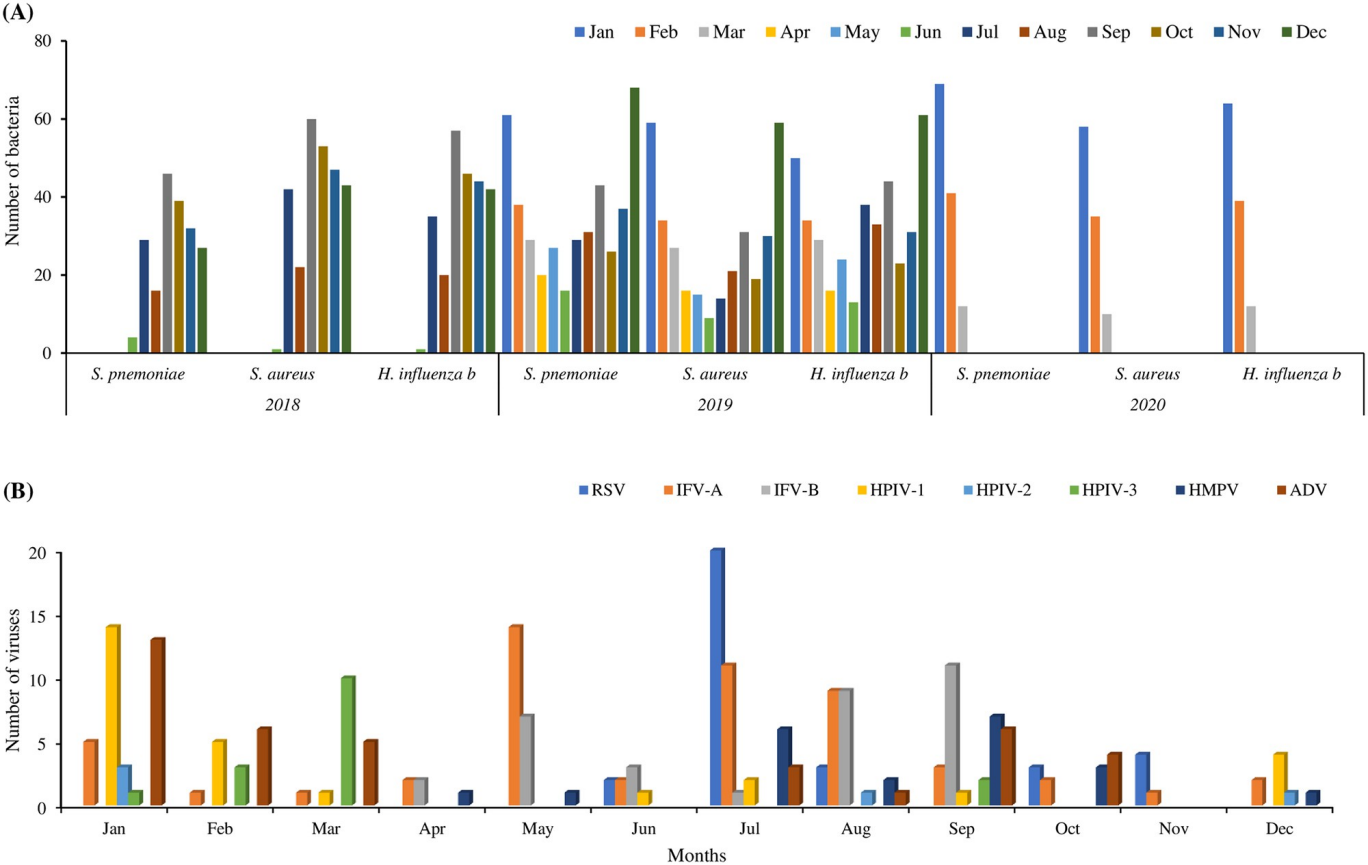
Seasonality of ARI pathogens in the study period. (A) Month-wise detection number of bacteria from NPS samples of ARI cases by qRT-PCR. (B) Month-wise detection number of viruses from NPS samples of ARI cases by qRT-PCR.

For all three major pathogens *S. aureus* (n = 60), *H. influenza b* (n = 57) and *S. pneumoniae* (n = 46) highest detection number reached the highest peak in September 2018. In 2018 case enrollment started in July when bacterial pathogens reached the highest peak in September followed by October and November. However, for the next two years in 2019 and 2020, the detection number for these three bacteria was highest during December (*S. aureus* 59, *H. influenza b* 61 and *S. pneumoniae* 68) and remained high between Septembers and February. In 2020, cases were enrolled until the month of March when January was the highest peak for the detection of all three bacterial pathogens. All eight viruses detected in the three-year period of the study are shown according to the month of sample collection. Low numbers of virus detections were seen compared to bacteria detection. However, RSV reached its highest peak in July (n = 20) and it was roughly constant for other months. Similarly, IFV-A detection was higher in May (n = 14) than in July (n = 11), IFV-B (n = 11) in September followed by August (n = 9). For HPIV-1 it was highest in January (n = 14) and HPIV-2 only 5 were detected, whereas 3 were in January. March was the highest time for HPIV-3 (n = 10) and HMPV was detected in highest number in September (n = 7) and then July (n = 6). ADV detection was highest in January (n = 13) and it was found in similar numbers for February (n = 6), March (n = 5), September (n = 6), and October (n = 4). Overall, the months of July, August, September and January, February, and March were the periods of most virus circulation found (Fig 10).

## Discussion

To our knowledge, this is the first study conducted on a representative fragile population, i.e., FDMNs, which focused on both bacterial and viral etiology for ARI case group along with their matched control groups. This study also examined the level of involvement of each etiological agent in the onset of LARI as well as the association between respiratory viral and bacterial infections, single and co-infection frequency and their correlation pattern, distribution of *S. pneumoniae* serotypes, seasonality of isolated pathogens and the enrollment of LARI cases. The purpose of the control group was to examine the presence of bacteria and/or viruses in healthy participants in comparison to those with LARI cases and figure out whether there were any associations/differences etiologically. The patient enrollment data at different times of the year, pathogen identification seasonality pattern of LARI, *S. pneumoniae* serotypes distribution infections will help with different preventive measures, like vaccination. Sample collection from participants was carried out from June 2018 to March 2020, with modest enrollment of ARI cases between July to February, and the peak being in October. Pre-monsoon (March–May), monsoon (June–August), post-monsoon (September–November), and winter (December–February) are the four seasons of Bangladesh, where pre-monsoon, monsoon and post-monsoon seasons are basically the rainy seasons [29]. However, in our study, post-monsoon to winter covered 80% of ARI case enrollment, where as post-monsoon (October) was the highest peak. Seasonal fluctuation information might be useful in raising awareness among the general public and health professionals such as physicians and nurses. During the peak ARI season, preventive measures such as personal and in-house sanitation and cleanliness, avoiding mass gatherings, frequent hand washing as well as sufficient ventilation, may be implemented particularly in crisis settings where these factors might play crucial roles. Our study described that the majority of cases were observed between July to February corresponding to the monsoon and winter season which is a commonly known period for respiratory illness [30, 31]. Moreover, many studies have shown that winter is the peak season for respiratory infections in Bangladesh [32, 33]. However, no specific patterns of seasonal distribution were observed for viruses during the study period. Our study findings suggested that the most frequently detected viral pathogens ADV, RSV, and IFV-A had no distinguishable seasonal pattern and were detected almost throughout the year. However, ADV was detected in higher numbers from September to February and reached its peak circulation in January. Also, RSV detection remained high from July to November and highest in the month of July. For human parainfluenza virus (HPIV), detection was higher from July to November, HPIV-1 from December to January, HPIV-2 from December to January and HPIV-3 from February to March. For influenza viruses, IFV-A and IFV-B were found from April to October, which is similar to the previous study in Bangladesh [33]. Overall, during the months of April, May, and June viral pathogen detection was relatively low and circulation was higher only for influenza viruses, which circulated following no specific pattern rest of the months. The findings on viral pathogen seasonality in the monsoon and winter are similar to earlier studies in Bangladesh on respiratory viral pathogen detection [32]. All three bacteria in our study were found circulating throughout the year, particularly from September through December, with the peak months being December and January. However, in 2018 all these three bacteria *S. pneumoniae*, *S. aureus* and *H. influenza b* peaked during September. Throughout the post-monsoon and winter, particularly in this FDMNs crisis settings in Cox’s Bazar, Bangladesh, especially for children may need the proper preventive measures against ARI. However, because this study was for a short period, a longer study period, as well as sufficient data, are required to support the seasonality of the infections. Multiple virus detection is possible using real-time polymerase chain reaction (RT-PCR), which is more reliable and quicker than viral culture [34] and for different bacteria detection [35–37]. In our study pathogen identification using qRT-PCR enabled identification of pathogen proportion from NPS samples as well as blood specimens both in cases and control group. A total of 538 ARI cases were enrolled in this study and majority of them were from children less than 5 years of age (59%). In terms of bacteria detection from NPS samples by qRT-PCR, similar proportions were detected both in cases and control (80% vs 82%). Also, no difference was observed for sex and age in cases and control. The ≤5 years age group was found to have a significantly higher proportion of bacterial detection both in cases and control group than >5 years (p<0.05). Virus detection was significantly higher in the cases group compared to the control group (30% vs 16%, p<0.05) similar to previous studies [38, 39]. Also, both age groups ≤5 years and >5 years were found in higher proportion in cases than control group (p<0.05 and p<0.05). In case group, ≤5 years showed higher proportion than >5 years (p<0.05) whereas in control group no difference was found between age groups. In addition, virus detection was found significantly higher for both male and female groups in cases than control (p<0.05). Considering co-infection, a higher proportion was found for ≤5 years age than >5 years in cases (p<0.05) where in control group no age-specific difference was observed. Among the bacteria identified in our study *S. pneumoniae* was found in highest percentage both in ARI cases (80%) and control (82%) group followed by *S. aureus* and *H. influenza b*. *S. pneumoniae* may cause disease, but a robust immune system and a healthy balance between local flora and invaders can help to clean it out. The host is frequently and persistently colonized by *S. pneumoniae* due to weak defensive mechanisms, which might eventually cause illness [40, 41]. The longitudinal study by Lipsitch et al. implied that children were reservoirs because of the length of carriage and colonization [42, 43]. Althouse et al. came to the conclusion that infants play a far smaller part in transmission than toddlers and older kids do, despite the fact that infants account for a higher percentage of carriage [43]. However, these findings indicate that more study is necessary to properly understand the direction of transmission. Moreover, in our study, of bacterial detection, no significant differences were observed between cases and control which is corroborated with previous study [44]. Age-specific distribution suggested that in both age groups (≤5 years and >5 years) *S. pneumoniae* was found as most predominant among the three bacteria both in cases and control group. However, among 3 major bacteria *S. pneumoniae* was seen as most predominant for ≤5 years of age group than >5 years (85% vs 65%, p<0.05) in cases where *H. influenza b* were detected in a similar proportion with no significant difference. Where *S. aureus* was found in higher proportion for >5 years age both in case and control. Similarly, the PERCH multi-country case–control study showed that these same respiratory viruses and bacteria were frequently linked to ARI. Furthermore, ADV and influenza viruses were found more frequently in controls in our study who had no history of ARIs in the 14 days prior to recruitment and had no respiratory symptoms seven days after recruitment, according to participant or family reports. The exception was for *S. aureus* where it was detected in higher proportion in >5 years of age group both for cases and control which may suggest the opportunistic infection for older people.

Moreover, no gender differences were seen in pathogen detection in cases or in control group suggesting that gender has no impact in the case of ARI infection. Similar results have been found in a previous study [45]. Viruses attributable to ARIs in our study were ADV in 6% of cases, influenza virus (IFV-A 6% and IFV-B 4%), RSV in 5% and HMPV in 4% of cases. These findings are similar to a recent study [44]. These findings are consistent with previous research, such as Shi et al who found that RSV, influenza virus and HMPV were all substantially linked to ARI [46]. Age distribution data showed that ADV is significantly higher among ≤5 years of age both in cases and controls than the other age group. RSV was found in a similar pattern where no significant differences were observed for other viruses in age distribution. So, age stratification revealed the highest-risk group where ≤5 years age group was found in a consistent pattern in case and control both for bacterial and viral infection, in line with previous studies for this age group [11]. Many types of organisms naturally colonize the nasopharynx. The majority of research focuses on bacteria, where sometimes the impact of viruses in the makeup of the respiratory microbiota is likely to be overlooked [47]. *S. pneumonia*, *H. influenza b* and *S. aureus* which was found in this study to be associated with controls where *S. pneumonia*, *S. aureus* were found in higher numbers in control and these are well-known contributors to acute respiratory infection. The fact that some of the microbes were identified more frequently in control does not rule out their involvement with ARI in some cases. Our study findings were similar to the PERCH study, where *S. pneumoniae* was detected significantly more frequently in controls than in patients. However, *S. pneumoniae* and *H. influenzae b* were strongly associated with lower respiratory tract infection [48]. However, a very low rate of blood culture positivity was found in our study, which is also similar to previous studies [49–51] and this finding may suggest bacterial pathogen identified from blood culture was only 5% hence indicating the true cause of infection rather than the carriage in the nasopharynx. However, antibiotic resistance was present for almost all the isolated pathogens from blood culture for different antibiotics (amoxicillin, ampicillin, chloramphenicol, erythromycin, oxytetracycline, ciprofloxacin, levofloxacin, doxycycline) where penicillin-G and cotrimoxazole were most predominant. *S. pneumoniae* was found resistant to penicillin-G, cotrimoxazole, and gentamycin. With the increased use of antibiotics, *S. pneumoniae* transforms and evolves, acquiring several genes for antibiotic resistance. Pneumococcus strains that are currently resistant to penicillin have spread throughout the world, and they are also resistant to other classes of antibiotics: tetracycline, chloramphenicol, and erythromycin corroborated with our study findings [52, 53]. In addition, in our study, qRT-PCR method was found more sensitive for bacterial detection (16%) including three bacteria (*S. pneumonia*, *S. aureus*, *H. influenza b*) from blood specimen following DNA extraction which may indicate the importance of RT-PCR method in terms of ARI pathogen detection, where a previous study also suggested the use of this technique [35, 37]. However, it is important to see how the presence of bacteria affects the co-infection, which may complicate the clinical symptoms and their management. We also analyzed co-infection data in terms of co-occurrences of at least two or more pathogens, where *S. pneumoniae* was found both in single and co-infection in a similar proportion, where co-infection was higher than single infection for *S. aureus* and *H. influenza b* were found both in cases and control group (p<0.05). For viruses, co-infection was higher for RSV, IFV-B, HPIV-3, ADV, and HMPV than single infection both in cases and control group. Where single infection was higher for the rest of the viruses. Moreover, the co-infection pattern is very important and can play an important role in causing disease and can alter the etiology. Our study suggested that the co-infection pattern is more common between bacteria and bacteria or viruses and bacteria but negligible numbers were found between viruses, as also found in a similar previous study [6, 54]. More specific analysis for correlation pairing between pathogens in case group showed the bacterial-viral synergistic effects were mainly from *S. pneumoniae* with other bacteria and viruses and the co-infection number was higher in ≤5 years of age. In our study, among the ≤5 years group highest co-infection number was found mainly from *S. pneumoniae* with *H. influenza b* followed by *S. aureu* s, RSV, IFVs, ADV. RSV was found with other bacterial pathogens in the highest number followed by ADV, IFVs, HMPV and HPIV-2. Number of co-infections was less in >5 years group than the other age group where all the viruses interact with *S. pneumoniae* to a similar extent except for HPIV-2 and HPIV-3 and correlation findings corroborated with the previous study. In the case group, the correlation pattern (positive and/or negative) was observed for bacteria and viruses for both age groups, whereas in the control group, no such correlation was found. In the case group, the correlation pattern of co-infection in ≤5 years age was different from >5 years age group. In ≤5 years of age, most predominant co-infection was between RSV and *S. aureus*, RSV and *H. influenza b*, RSV, and ADV where the correlation was positively correlated and a negative correlation was observed for RSV and IFV-A. Similarly, a previous study showed when RSV infection rates are high, influenza infection rates are low due to viral competition, and vice versa [55]. In >5 years age, the numbers of co-infection between bacteria and viruses were higher than ≤5 years group. Positive interactions were between influenza viruses and *S. aureus*. Also, *S. pneumoniae* was positively correlated with ADV, HMPV and HPIV viruses. However, no positive correlation was observed between bacteria where they were negatively correlated, suggesting that *S. pneumoniae* less likely to coexist with other bacteria rather than viruses. Also, some previous studies suggested *S. pneumoniae* colonization may enhance during viral co-infection and increased colonization during viral infection may induce the transmission [56, 57]. Moreover, very few numbers of co-infection were detected between viruses (RSV and HMPV) where a higher number of bacterial-viral infections may correlate with the previous findings and their proposed mechanisms where a wide range of pathogens interact with viruses through resource competition, immune response or interaction between viral protein, etc. [58, 59]. Overall, co-infection pattern and their number may suggest their interaction and that they can facilitate secondary infection for viruses or bacteria and this phenomenon is common in respiratory infection [60]. Our current findings may suggest, either bacterial or viral infection in combination with either pathogen can trigger secondary optimistic infection, which perhaps suggests future research. Moreover, there is growing evidence suggesting an association between bacterial colonization and future ARI occurrence [61], which is related to our bacterial interaction findings. In addition, viral infection may enhance bacterial superinfection by promoting bacterial attachment sites on nasopharyngeal epithelial cells and increased mucous production may further enhance bacterial growth [62]. Also, sub-clinical infection in asymptomatic cases may act as a source of pathogen transmission [44]. Furthermore, a logistic regression analysis showed that case groups were more likely to be positive for RSV four times, HPIV-3 four times, HMPV twenty times and IFV-B two times more than the control group. Moreover, ≤5 years group was likely to be positive for RSV, HMPV, and IFV-B in a higher percentage than the other age groups. Prior investigations of bacterial and viral loads in the upper respiratory tracts of young Congolese children suggested the importance of the load pathogens [63] and to our best knowledge there is no study conducted among the FDMNs focusing on the viral and bacterial load in ARI cases and healthy individuals. Cycle threshold (Ct) values are continuous, semi-quantitative assessments of viral load used in qRT-PCR [63, 64]. However, our study evaluated the bacterial and viral prevalence according to Ct value detected by RT-PCR where the detection number was higher for all the bacteria considering Ct≤30 in the case group than the control group. However, the percentage was higher in control group when a lower load (Ct>30) was considered. In addition, viral load (Ct≤30 and Ct>30) was found always high in case group for both the age (≤5 years and >5 years) than the control group for all the viruses except for ADV and HMPV-1 corroborated with previous study [65]. However, understanding the complicated interactions between viruses, bacteria, and viral–bacterial interactions could contribute to the understanding of respiratory pathogen epidemiology and planning public health strategies. *S. pneumoniae* is the leading cause of child mortality especially for younger children of ≤5 years despite the vaccination program and it is responsible for 33% death worldwide [66]. Some of the discrepancies were most likely due to methodological differences, whereas others were most likely due to actual demographic disparities. Bangladesh and different NGOs introduced PCV-10 vaccination among the FDMNs and current PCV-13 replaced previous PCV-7 and PCV-10 which is protective against 13 serotypes [53, 67]. In our study, we identified 40 serotypes from NPS using RT-PCR where few serotypes/serogroups were found in higher proportions 6AB, 14, 23F, Sg18, and 5. Serotypes/serogroup 6AB (12% and 13%) and 23F (10% and 9%) were found in higher proportion under PCV-10 in cases and control. However, serotypes 16F, 35B, 15A, 20, and 10F were predominant in case group whereas 11A, 10A, 34, 35A, and 13 were predominant in control group in higher proportions. Among the nonPCV-10 serotypes/serogroups higher proportion (>5%) were found for 19A, 11A, 16F, 35B, 15B/C, 15A, 20, 10F both for the case and control groups. Even if the 10- or 13-valent vaccines now being developed are only effective against half of the strains that cause invasive pneumococcal illness, their introduction into the huge number of FDMNs population might be beneficial. However, there are a number of strains that aren’t covered by the current vaccine candidates. Here, several co-infection patterns and their positive/negative interaction between 41 serotypes and all other pathogens tested might help in vaccination strategy planning in the future. Correlations between viruses and serotypes were observed in higher numbers than the bacterial interactions with serotypes which are predominant in cases over the control group. Overall, this study suggests that *S. pneumoniae* is the most predominant bacteria followed by *S. aureus* and *H. influenza b* in ARI patients. Among the viruses, RSV was the highest detected followed by ADV, IFVs and HMPV, and their seasonal pattern was mostly during rainy monsoon and cold dry winter seasons in Bangladesh. Age group distribution showed ≤5 years age was found as the highest risk group. Moreover, single and co-infection were found similar for *S. pneumoniae* where the co-infection number is significantly higher for *S. aureus* and *H. influenza b*. For viruses, RSV, HMPV, IFV-B and HPIV-3 were found in high proportions for co-infection than single infection, both in cases and control group. Also, the co-infection pattern showed the predominant interaction occurred between bacteria and viruses and very limited extent for virus and virus. Besides, high viral and bacterial loads were present in the ARI case group compared with the control for most of the pathogens.

However, this study has several limitations, like it was not possible for us to collect sputum samples since most of the cases were children. Also, in the context of this study, X-rays were not available and decisional tree for disease presentation was established based on clinical symptoms and examination findings only as our study was in such humanitarian crisis settings. Also, only very severe cases were hospitalized as per suggestion by the physician or referred to the tertiary hospitals. Our study did not follow up with the participants for tertiary hospitalization. Moreover, our study period was not long enough to determine the actual seasonality of ARI pathogens. Besides, we were unable to differentiate the *S. pneumoniae* serotypes like 6A and 6B, 15B and 15C, 9N and 9L or detect serogroups 18 and 24 because of the limitations of the RT-PCR method we used. Despite this limitation, we observed ARI etiology related to age, gender, single and co-infection pathogen spectra, *S. pneumoniae* serotype distribution pattern, which may help in public health strategies like identifying the predominant respiratory pathogen, identifying the highest risk age group, and vaccine administration in such humanitarian settings.

Importantly, the prevalence of viral infections data may play a crucial role in preventing the unnecessary use of antibiotics. Also, *S. pneumoniae* serotype distribution data may also be useful in determining disease-causing serotypes and future vaccine development.

## Conclusion

Overall, our study on the FDMN population found that viral pathogens such as ADV, IFVs, and RSV were the most commonly detected pathogens in the ARI cases. Co-infection incidence was higher among the case group than the control group for viruses including RSV, IFV-B, and HMPV. The detection of bacteria was comparable between the two groups, with the highest detection observed for *S. pneumoinae*. Both bacterial and viral pathogen detection rates were higher in children ≤5 years of age. Moreover, a higher prevalence of *S. pneumoniae* serotypes including 19A, 6A/B and 23F within the PCV-13 vaccine where 13, 15B/C and 35B serotypes within non-PCV-13 were observed. *S. pneumonia* e serotype distribution as well as their infection pattern with other pathogens in cases and controls provide crucial information for public health policy like vaccination strategies in such crisis settings. The study also suggests that the patterns of co-infection with different pathogens may play a role in disease progression, however, further investigations are required to fully understand the mechanisms and consequences of such conditions.
